# Medical Items and News of Interest to the Physician

**Published:** 1872-04

**Authors:** 


### Medical Items and News,

### For the Use of Physicians.

CULLED FROM THE STANDARD MEDICAL JOURNALS OF THE WORLD.

NOTE.—These formulæ are intended only for the regular practitioner of medicine, and should be employed only by him, or under his immediate supervision.

**Carbolized Glycerine.**

Glycerine 100 parts; carbolic acid, 1 part. Mix. For impetico, chronic eczema, lichen, prurigo, and pemphigus.—*New Remedies.*

**Incontinence of Urine in the Aged.**

Dr. Schmidt recommends one drop of the tincture of iodine every two hours for this trouble. He has found marked relief following its use.—*New York Med. Journal.*

**Gonorrhœa.**

A writer in the London *Lancet* claims to cure gonorrhœa and gleet in from two to six days, by injecting a solution of permanganate of potash, five to ten or fifteen grains to the ounce of water. It causes no pain or inconvenience.

**Dyspepsia.**

Dr. Warren of Boston, (*Journ. Gynæcological Soc.*), has employed, with great benefit, the sulpho-binate of sodium in dyspepsia, especially that accompanying pregnancy. He gave it in doses of eight grains, three times a day.

**Strychnia Poisoning.**

Dr. L. B. Kay, Chillicothe, Mo., (*Kansas City Med. Jour.*), records two cases of this character which were relieved by the inhalation of chloroform for eight hours, when the use of bromide of potassium and morphine was begun.

**Atropia in Opium Poisoning.**

Dr. Dan'l. S. Bucklin, Lansingburg, N.Y., (*N. Y. Med. Jour.*), records two cases of opium-poisoning, successfully treated by atropia. He ordered Fleming's solution of atropia in the minimum (20 drops) doses.

**Traumatic Tetanus.**

Dr. P. H. Spohn, of Penetanguishene, Canada, (*Canada Lancet*), has used the spinal ice-bag in traumatic tetanus, and is convinced that it will not prove a "panacea," as has been advocated by Dr. Chapman and others. Although he did not accomplish what he expected, still it gave more prominent relief than anything else. He found that the local application of tobacco with the ice-bag was better than the ice alone.

**Suppositories in use with Pessaries.**

Dr. Ephraim Cutter, of Woburn, Mass., (*Jour. Gynæcological Soc.*), in an excellent article on retroversion of the uterus, advocates suppositories of iodoform, grs. ij. to grs. x. cocoa butter, before or after the use of the pessary, they being useful to allay vaginal irritation.

**Dressing for Burns.**

Mr. Robert L. Snow says of oakum as a dressing for burns, that it induces the cicatrization of burns with remarkable rapidity; it induces healing action in those indolent ulcers which are the result of defective hygienic conditions; it prevents all smell; it is cheap, saves time and trouble, and the resulting cicatrices do not contract.—*British Med. Journal.*

**Amputation of Uterus.**

Dr. Thos. Hay, of Phila., publishes an account of a successful amputation of an inverted uterus with an intra-fibrous tumor, by means of ecrasement. The patient made a rapid recovery and regained good health. An account of the operation may be read in number 770 of the Phila. *Med. & Surg. Reporter.*

**Albuminuria.**

Brignoli, in *Lo Sperimentale*, besides recommending nux vomica in various neuroses, gastralgia, dyspepsia, cardiac palpitations, periodic cough, etc., states that he has observed it to have a marked effect in retarding the progress of albuminuria, especially the scalatinal form with anasarca. He cites twelve cases of complete recovery.

**Epilepsy.**

Prof. Meredith Clymer recommends the use of bromide of sodium in epilepsy, instead of bromide of potassium. He thinks the latter, when given for a time in large doses, brings on serious disturbances of the mental faculties and muscular feebleness of the lower extremities. The bromide of sodium is free from these objections; is more readily absorbed and eliminated, and has a less depressing influence upon the heart.—[*Extract from Report of Clinic at Albany Med. College.*]

**Diabetes.**

Dr. G. Moore, of Hastings, (*British Med. Jour.*), lately read a paper on a case of diabetes of three months' standing. The patient had previously been rigorously dieted. Under a bread-and-milk diet, (three pints of the latter daily) and the administration of effervescing salines, with iron, the urine became perfectly free from sugar in a fortnight, and the patient speedily gained flesh and strength. Dr. Moore expressed himself strongly in favor of the free use of milk in such cases.

**Otitis.**

In the proceedings of the Lowndes County Medical Association of Miss., we notice that Dr. Brownrigg recommended tobacco as an infalliable remedy for otitis. The following is his formula;

℞

Tobacco, (cut fine) ʒ j;

Glycerine, ℥ j. M.

Sig:—Place five drops in the ear, confining it with cotton, once a day.

**Acute Glanders in a Man.**

Dr. Tuske reports in the *Wiener Mediz. Presse*, Dec., 1871, a case of glanders occurring in a man. After Alkaline bath and the internal administration of quinine proved of no effect, six grains of carbolic acid in solution were ordered to be taken daily. Under this treatment the patient recovered, and was discharged from the hospital on the thirty-fifth day.

**Asthma.**

This prescription is particularly recommended in cases of asthma, by J. Hale, M. D., of Owensborough, Ky., (*American Practitioner*):

℞

Ether sulph., ℥ i ss;

Tr. lobeliæ, ℥ j;

Tr. opii,

Tr. stramonii, āā ʒ iv. M.

Sig: Teaspoonful every hour or two until the dispnœa is relieved.

**Chloral in Cholera.**

Dr. Von Reichard has employed chloral in the recent epidemic of cholera in Riga. He used it first, to calm the cramps; secondly, to lessen the præcordial anguish in the last stage; thirdly, to arrest the vomiting; fourthly, to induce sleep. It has successfully fulfilled all these indications. He relates one case in which the patient was *in extremis*, and had apparantly not three hours to live; sixty grains of chloral gave calm sleep; the temperature rose; the pulse fell from 130 to 90 and regained its fullness; the *facies cholerica* disappeared; and the patient recovered. Dr. Blumenthal also, in three cases of severe cholera, saved two, by means of chloral. The doses given were sixty grains in half ounce of water, twice or thrice an hour.

**Carbolized Gargle for Diphtheria.**

Charles Sedgwick, Jr., (*New Remedies*), recommends the subjoined formula:

℞

Carbolic acid, ♏ xx;

Acetic acid, ʒ ss;

Honey;

Tincture of myrrh, āā f ʒ ij;

Water, to f. ℥ vj.

The acids to be shaken together well before the other ingredients are added.

**Malarial Fevers.**

Dr. A. R. Brissett, of St. Johns, N. B., (*Canada Lancet*), writes that the following formula is a useful one, where quinine alone has failed in cases of intermittent and remittent fevers:

℞

Ferri subcarb., ℥ ss;

Quiniæ sulph., ℈ iss;

Syr. simplex, ℥ vj. M.

Sig: A teaspoonful four times a day.

**New Treatment for Encysted Tumors.**

Dr. A. J. Perkins says, in the *Georgia Medical Companion*, that the hypodermic syringe is an effectual instrument in the cure of those encysted tumors of the scalp usually called wens. It is to be charged with pure tincture of iodine and the point inserted well into the body of the tumor, and as much of the tincture forced in as the tumor will contain. In a few days the sac sinks away from the surrounding parts, and may then be easily removed.

**Goitre.**

At a recent clinic conducted by Prof. Gross, he recommended the following treatment in a case of large goitre, in an unmarried woman, aged 26. Wash the neck at least once in the 24 hours, with hot water and soap, and immediately afterwards, a portion of the following ointment should be applied to the surface oı the tumor, and well rubbed in:—

℞

Ung. hydrarg. biniodid., ʒ j

Cerat. simp., ʒ vj. M.

Have the patient also take internally the *liquor iodinii compositus*, gtt. viij. in sweetened water, three times daily. A piece of flannel and oiled silk must be worn about the neck.—Avoid all red meats, and have the patient take six grains of blue mass, in combination with a grain of ipicac, once a week.

**Tapeworm.**

Dr. S. R. Knight, of Phila., (*Phila. Med. Times*), successfully treats cases of tapeworm by kameela. In the case of a patient, aged fifty-six, it was given according to the following formula:

℞

Kameelæ, ℥ ss;

Syrp. simpl., f. ℥ j; M.

Sig; Take a tablespoonful at a dose. After one dose, a worm fifteen feet in length was expelled.

**Anasarca.**

Dr. J. Hale, (*American Practitioner*), has used the subjoined prescription in several obstinate cases of anasarca with complete success:

℞

Tr. digitals, ℥ j;

Potass. iodid., ʒ iij;

Vin. colchi. rad., ʒ ij;

Syrp. sarsap., comp., ℥ iij;

Aquæ puræ, ℥ ij. M.

Sig: A teaspoonful three or four times a day. The bowels to be freely moved every three or four days with pulv. jalapæ comp. He says that it is very valuable in other forms of dropsy.

**A New Mode of Giving Copavia.**

Dr. J. H. Wehner, of Germantown, Pa., writes to the Phila. *Med. & Surg. Reporter*, that he has obtained the best therapeutical action of copavia with the entire absence of its nauseating and other disagreeable effects, by using it in the form of a suppository. His formula is as follows:

℞

Copabiæ, f. ℥ vj;
Opii pulvis, gr. vj;
Olei theobromæ,
Cetacei, āā ℥ jss;
Ceræ albæ, gr. xlv. M.

Misce secundum artem et fiat suppositoria, No. xij.

Sig: One to be introduced into the bowel morning and night. If constipation occurs, administer moderate dose of Rochelle salts.

**Scabies.**

The following formula is extolled for itch, by Dr. W. C. Moore, of Atlanta, Ga., (*Georgia Med. Companion*):

℞

Simple cerate,

Sulphur, āā ℥ j;

Bicarb. sodœ, ℥ ss;

Ungt. Hydrag., ʒ j. M.

Sig: Smear a small quantity over the affected parts every night. Wash well with soft soap and water before each application.

---

**Management of Dysentery.**

J. Hale, M. D., of Owensborough, Ky., (*American Practitioner*), reports some of his favorite prescriptions, which he has found to be useful in cases under his observation.—The following formula, for dysentery, was introduced in that section by Dr. Murray, an eminent practitioner, twenty-five years since, and has been in general use ever since by many Kentucky physicians:

℞
Sodæ sulph., ℥ j;
Morphiæ sulph., gr. j;
Aqua pura, ℥ vj. M.

Sig: Tablespoonful every two hours until free watery operations are produced, then prolong the interval to four hours, and continue until the dysenteric discharges, pain, tenesmus, etc., ceases.

---

**Intermittent Fever.**

Dr. Treulich (*Bost. Med. & Surg. Journal*), publishes eight cases of intermittent fever promptly cured by carbolic acid. His formula is:

℞
Acidi carbolici, gr. iij;
Inf. gentian, ℥ v;
Syrp. simpl., ℥ ij. M.

Sig: One drachm ter die.

Carbolic acid, he affirms, is an admirable remedy for intermittent fever, even for obstinate cases which have resisted quinine. Its action is speedy and certain, and it requires such a small amount that it cannot possibly have any injurious effect upon the system.—It costs only one-thirty-fifth as much as quinine, and for this reason is to be preferred for the poor. This successful use of carbolic acid proves to him that the action of quinine in intermittent fever is antiparasitic. It also favors the opinion that the fever is the result of blood poison.

**Strychina Hypodermically.**

Dr. R. A. Vance, of N. Y., (*Medical World*), says that the solution of strychina for hypodermic medication, which his experience has taught him to consider best, is one containing a grain of the drug to one drachm of water; the solution being effected, by the addition of a small quantity of dilute acetic acid. His formula is as follows:

℞

Strychniæ sulph., gr. j;

Acid acet. dil., ♏ j;

Aquæ ad, ʒ j. M.

S. Ft. Sol.—One minim contains 1 60 of a grain of strychnia.

---

**Management of Epilepsy.**

Dr. Brown-Sequard recommends in the treatment of epilepsy, the following combination of the bromides of ammonia and potassium:

℞

Potassii iodidi, ʒ j;

Potass. bicarb., ℈ ij;

Potassii bromidi, ℥ j;

Ammonii bromidi, ʒ ij ss;

Inf. columbæ, ℥ vj. M.

Sig: A teaspoonful before each of the three meals, and three teaspoonsful at bedtime, in a little water.

To prevent the development of bromism, he is in the habit of combining arsenic with the bromide of potassium. Since using this combination, has not observed so much debility caused by its prolonged administration.

The use of iron, strychnia, the hypophosphites, is also indicated to maintain the health of epileptics during a course of bromide of potassium. The hygienic means consists of abundant food, wine, out-door employment, and a careful regulation of the moral life.—*Medical Record.*

---

**Tænia Solium in Child Five Days Old.**

Dr. Samuel G. Armor reports, in the *N. Y. Med. Jour.* for December, the case of a child in whom trismus and other nervous symptons were found to be dependent upon the presence of tape-worm in the intestinal canal. When the child was five days old, a grain of calomel was given to it for the relief of these symptoms, and about ten hours afterward the child passed two segments of the parasite. Anthelmintics were then administered, which caused the expulsion of numerous other segments, with entire relief to the symptoms. Some of the segments were examined microscopically by Dr. Segar, who reported that they unquestionbly were part of a tænia. Two months after the birth of her child, the mother, who did not suspect the presence of tape-worm, and who was apparently in good health, was put upon treatment for tape-worm, taking, while fasting, the emulsion of pumpkin-seed. At the end of twenty-four hours she passed over seventy segments of tænia.

**Epileptic Vertigo.**

Dr. S. L. Raines, of White Plains, Tenn., in a letter to the Phila. *Med. & Surg. Reporter*, gives the history of a very interesting case which he treated successfully in the following manner;

℞

Ext. hyos.;

Quiniæ,

Iodoform, āā ℈ ij;

Ext. nucis vomicæ, ℈ ss;

Hydrocyanate of Iron, gr. xv;

Leptandrin, ʒ ij.

Misce, et fiat massa in pillula 80 dividendum.

Sig: Two pills, three times daily, after meals.

Also:—

℞

Pot. iodidi, ʒ j;

Pot. bromid. ʒ ijss;

Ammon. bromid., ℈ij;

Vin. pepsinæ, f. ℥ iv;

Aquæ, f ℥ ij; M.

Sig: A desertspoonful before each meal, and two before going to bed.

**Singular Accident.**

A hack driver applied to us recently with one eye entirely closed by reason of an immense swelling of both eye-lids. He had just received a blow from the "gamble-joint" of a horse, which produced no serious pain, but immediately afterward, in blowing his nose, the eye closed in the manner described. We found the swelling to be caused by an accumulation of air in the subcutaneous-areolar tissue, and having punctured it with a small trocar, the air at once passed through the canula, and all traces of the "swelling" disappeared. We directed him to hold his nose and again force air through the passage, when the immense distention again occurred, puffing up both lids as large as a hen's egg. The lacrymal sac must have been ruptured by the accident, through which the air found passage, from the nose into the cellular tissue. We have never read of a similar case, and for this reason record it.—EDITOR BISTOURY.

**Antidote for Rattlesnake-Venom.**

Dr. R. Stern, of Fredericksburg, Texas, in an article to the *Med. & Surg. Reporter*, gives some interesting experiments with the venom of the Rattlesnake. His experiments go to prove that the venom of the snake given as a medicine, is a sure antidote to its poison when communicated by the bite of the reptile. The following is his formula:

℞
Fel. crotali horridi, gtt. j;
Spiritus vini rectificati, gtt. x.

Misce, bene agitando. Dose.—Place five drops in a tumblerful of water, of which give an adult a tablespoonful every five to fifteen minutes.—The Doctor says he gave a bitten rabbit teaspoonful doses. After the third dose the convulsions ceased; after the fifth he did not look so anxious, and got up; after the tumblerful of the dilution, containing one drop of the venom, had been given, the rabbit had entirely recovered.

We should call this good homœpathic treatment, for the publication of which our brethren of that faith should feel obliged.

**Epilepsy.**

In the *Wien. Med. Zeit.* of recent date, we find an article from Dr. Voisin who gives the treatment adopted by the late Herpin, who was so successful in curing epilepsy. Dr. Herpin was unfamiliar with the use of bromide of potassium in the treatment of epilepsy, and Dr. Voisin has no confidence in it in this disease. Herpin treated his patients chiefly with lactate of zinc, ammonio-sulphate of copper, hyoscyamus, and artimisia. In a young girl, aged 13, who had contracted essential epilepsy, he treated her ten years later, after she had had 111 attacks of *grand mal*, and numerous fainting fits and vertigo. After the patient had been treated with lactate of zinc, hyoscyamus, and artimisia, she was cured when the last remedy was raised to 10 grammes. The treatment was continued one year longer, and resulted in a complete cure.

The second patient was 21 months old when brought to Herpin; the disease being heredi-

-tary and fully developed. The attacks came on 25 times a month, and one hundred and fifty had occurred. Treatment consisted in lactate of zinc, ammonio-sulphate of copper, hyoscyamus and artimisia. The disease became milder under use of the copper, and disappeared entirely when artimisia was used, and since 1858 the child, who was now a man, was quite healthy.—Numerous other cases are given, but the above are sufficient to attract the attention of the profession to the remedies used.

**Spermatorrhœa.**

Prof. Gross at his clinic Dec. 24, recommended the following treatment:

Patient ordered to dash cold water against his genitals before getting into bed; to sleep upon a hard mattrass, and to take internally:

℞

Elix. cinchonæ calis. ʒ iss:

Acid nit. dil., gtt. viij;

Strychniæ sulph., gr. 1-16. M.

Sig: Take the above quantity 3 times daily.

Also:

℞
Morphiæ sulphatis, gr. ¼
Cocoa butter, q. s.
Fiat in suppository, j.

Sig: Introduce into the bowel at bedtime.

Also:

℞

Liquor plumbi subacetas, ʒ j;

Aquæ, f. ℥ x. M.

Sig: Inject into the urethra twice in 24 hours.

**Re-Vaccination.**

Much is being said and written upon this subject, and as it is particularly interesting at this time, we give the opinion of Prof. Wood, as laid down in his "Practice of Medicine":—

"This operation should be performed in every case which has not been tested by exposure to small-pox contagion during an epidemic prevalence of the disease. It may be asked whether vaccination should be employed in persons previously affected with the small-pox. I should unhesitatingly answer this question in the affirmative. It has been before stated that, though fewer persons are attacked with varioloid after inoculation or natural small-pox than after vaccination, yet a great number perish.—The same protection that a second vaccination extends in one case will probably be extended by vaccination in the other, and is even more needed, at least so far as life is concerned. It is generally stated in the books that vaccination after small-pox produces little or no effect. My own observation has been exactly the reverse. In concluding this subject, I would again strongly urge the propriety of universal vaccination, as the means not only of promoting the comfort and possibly of saving the life of the individual, but also of preventing the spread of small-pox and of ultimately eradicating it, if not from the globe, at least from extensive communities."

**Diseases of the Ear.**

The following practical hints are taken and condensed from the new work of Dr. Lawrence Turnbull, on diseases of the ear:

FOREIGN BODIES IN EAR can almost always be washed out to the orifice by means of gentle syringing with warm water, and can then be removed by the angular forceps.

ECZEMA AURICULÆ is treated in its acute form, by soothing applications of bland fluids, the ear being covered with oil silk, and tonics administered internally. The chronic form of the disease is treated with ointments of the protonitrate of mercury, or the benzoated oxide of zinc.—Internally, *Fowler's solution*, or the solution of the bichloride of mercury.

INSPISSATED CERUMEN should be broken up and removed by the curette, but if hard it must be softened by a warm solution of bichromate of soda, in rose water (gr. j to f. ℥ j), and then removed by the syringe or curette.

FURUNCULOUS ABSCESS is most excrutiatingly painful, slow in development, and forms a core which is discharged with difficulty. There exists also a plugging up of the meatus by a swelling of its tissues. The most successful treatment has been the application of moist warmth to the parts, and a free incision into the abscess, as soon as its presenting point could be determined.

ACUTE AURAL CATARRH of the middle ear, with perforation, is treated by keeping the parts cleansed by Clark's or Thudichum's douche, with astringents, tonics, and counter-irritation, with occasional depletion by leeches or small cups.

ACUTE INFLAMMATION OF MEMBRANNA TYMPANI.—This affection is accompanied with violent pains in the ear, occurring suddenly, with more or less fever and suppuration. Meatus dry, membrana tympani much inflamed, sensative, opaque, dull, flat and thickened. Treatment:—Perfect rest, free leeching, injections of warm water, and application of glycerine and opium; and toward the termination, mild astringents, counter-irritation and use of air-douche.

